# Audio-visual feedback improves the BCI performance in the navigational control of a humanoid robot

**DOI:** 10.3389/fnbot.2014.00020

**Published:** 2014-06-17

**Authors:** Emmanuele Tidoni, Pierre Gergondet, Abderrahmane Kheddar, Salvatore M. Aglioti

**Affiliations:** ^1^Department of Psychology, Sapienza University of RomeRome, Italy; ^2^IRCCS, Fondazione Santa LuciaRome, Italy; ^3^CNRS-AIST Joint Robotics Laboratory, UMI3218/CRTTsukuba, Japan; ^4^UM2-CNRS LIRMM UMR5506Montpellier, France

**Keywords:** brain computer interface, SSVEPs, sense of agency, humanoid, teleoperation, motor control

## Abstract

Advancement in brain computer interfaces (BCI) technology allows people to actively interact in the world through surrogates. Controlling real humanoid robots using BCI as intuitively as we control our body represents a challenge for current research in robotics and neuroscience. In order to successfully interact with the environment the brain integrates multiple sensory cues to form a coherent representation of the world. Cognitive neuroscience studies demonstrate that multisensory integration may imply a gain with respect to a single modality and ultimately improve the overall sensorimotor performance. For example, reactivity to simultaneous visual and auditory stimuli may be higher than to the sum of the same stimuli delivered in isolation or in temporal sequence. Yet, knowledge about whether audio-visual integration may improve the control of a surrogate is meager. To explore this issue, we provided human footstep sounds as audio feedback to BCI users while controlling a humanoid robot. Participants were asked to steer their robot surrogate and perform a pick-and-place task through BCI-SSVEPs. We found that audio-visual synchrony between footsteps sound and actual humanoid's walk reduces the time required for steering the robot. Thus, auditory feedback congruent with the humanoid actions may improve motor decisions of the BCI's user and help in the feeling of control over it. Our results shed light on the possibility to increase robot's control through the combination of multisensory feedback to a BCI user.

## Introduction

Walking through an environment, avoiding possible obstacles and stopping close to a desired place and act upon objects are motor decisions that people without any physical impairment can easily plan and quickly achieve in various environments.

Current research in Brain-Computer Interface (BCI) with electroencephalogram (EEG) shows the successful use of mobile robots to accomplish complex tasks (Bell et al., 2008; Millán et al., 2010; Choi and Jo, 2013). Nonetheless the possibility to achieve a natural control of a real size humanoid robot within a real environment (Gergondet et al., 2013) still represents a great challenge for computer science, neuroscience, and BCI research. Such challenge is well illustrated by walking tasks that are typically considered comparatively simply. However, although seemingly simple, walking behavior is the outcome of a long-lasting learning process, involves motor and intellectual skills and generates sensory consequences we give very little attention to. For example, under normal circumstances we barely pay attention to the sound generated by our footsteps, possibly due to their high predictability. However, imagine hearing footsteps not directly related to your own walk (i.e., a sound that does not match the instant of hit of your foot with the ground). It is highly likely you start thinking something unusual is happening to you or that someone is following you. Footsteps sounds are directly related to our walking behavior and represent a sensory feedback that also informs about the agent of the action. The present study aims at assessing the importance and benefits of accurate auditory feedback relative to footsteps sounds during a BCI-based steering of a humanoid robotic surrogate.

The feeling of being in control of one's actions and/or its consequences is called sense of agency (SoA, Gallagher, 2000; Synofzik et al., 2008; David, 2012). Importantly SoA is a multifactorial feeling (David, 2012) and can be manipulated by altering temporal predictability between an action and its effect (Sato and Yasuda, 2005). In particular, increasing the temporal interval between an action and its effect decreases the perceived SoA. Relevant to this study is the SoA over footstep sounds. In a study conducted by Menzer et al. (2010), participants were asked to indicate whether a footstep's sound was self-produced or not. More specifically follow a path while hearing through headphones a footstep sound either synchronous or asynchronous with their real steps. The results show that SoA was significantly higher in the former than the latter condition indicating the temporal mismatch between actions and hearing the footstep sounds influences the feeling of being the cause of an action's consequence.

BCI systems typically need a time window to decode reliably the intended action and actually implement it. Time segments for data analysis change according to the type of EEG signals the BCI relies on and the method that is used to process them. For example, to correctly classify the user's intention motor imagery- (MI), steady-state visually evoked potentials- (SSVEPs), and P300-BCI one needs 4 s (Guger et al., 2003), 7 s (Guger et al., 2012), and 45 s (Guger et al., 2009; see also Table 2 in Guger et al., 2012 for comparative purposes), respectively.

MI-based BCI devices can have a maximum of four outputs (right and left arm, foot and tongue movement imagery, Naeem et al., 2006; but see Friedrich et al., 2013 for different mental strategies). Yet, a high level of accuracy is achieved only after lengthy period of training (Pfurtscheller et al., 2006; Onose et al., 2012). P300-based BCI systems can have a higher number of outputs (Guger et al., 2009) and require less training. However, these systems implies large and variable time windows (that range from 5 s for a 4-choice system (Bell et al., 2008) to 45 s for 15 choices (Guger et al., 2012). In the present study we used a SSVEPs BCI interface as the best compromise between the number of outputs, the training duration, and the required time window to classify user's intention (see Materials and Methods). We assessed BCI users' performance and subjective experience during a continuous whole-body control of a humanoid robot (HRP-2). In particular participants (located in Italy) remotely controlled through BCI a humanoid robot (located in Japan). A pick-and-place scenario (Gergondet et al., 2013) was adopted. The video from robot's cameras was fed back on a 2D screen. During the task, participants could observe or not HRP-2's body through a mirror and could hear a footstep sound that matched (synchronous) or not (asynchronous) the actual robot's movements (see Materials and Methods). In this way we tested the role of audio-visual feedback in an ecological scenario measuring the BCI users' performance (expressed by the time to complete the task) and their perceived quality of the interaction with the robot by means of a questionnaire (see section Quality of the Interaction).

We expected faster walking time when audio-visual feedback is congruent (synchronous condition) relative to when is not (asynchronous condition) and better precision in dropping the object when the robot's body was visible through a mirror (mirror condition) relative to when it was not (no-mirror condition).

## Materials and methods

### Participants

A total of 28 healthy subjects took part in the study. Nine subjects (3 women; age range, 20–26 years) participated in the main experiment and 19 subjects (12 women; age range, 21–33 years) were tested in one of the two pilot studies. All the subjects were right-handed according to a standard handedness inventory (Briggs and Nebes, 1975), had normal or corrected-to-normal visual acuity in both eyes, and were naive as to the purposes of the experiments. None of the participants had any contraindication for the BCI study (Fisher et al., 2005). Participants provided written informed consent and the procedures were approved by the ethics committee at the Fondazione Santa Lucia and were in accordance with the ethical standards of the 1964 Declaration of Helsinki. Participants received reimbursement for their participation and were debriefed on the purpose of the study at the end of the experimental procedure. In the SSVEPs-BCI experiment no discomfort or adverse effects were reported or noticed.

### Pilot studies—footsteps sounds selection

Two independent groups of subjects have been tested in different pilot studies (Group 1, 12 subjects, 7 female, range 21–33 years; Group 2, 7 subjects, 5 female, range 20–23 years). In Pilot 1, eight different human footsteps audio files were interleaved by a variable number (min 5, max 10) of pure tones to avoid habituation and randomly heard by participants. Subjects were asked to guess what the sound was and type the answer within a text box. Participants listened to each sound only once. The sound represented two “hits” with the ground (e.g., right-left foot). In Pilot 2 participants rated on a 0–100 Visual Analog Scale (VAS) how much the sound they heard was reproducible by the human body. The sounds were the same as in Pilot 1 and interleaved by pure tones. The selected sound was freely categorized as “footstep” by 91% of the sample from Pilot 1 and rated as reproducible by the human body at the 93.7 ± 2.57 (mean ± s.e.m.) on the 0–100 VAS scale from Pilot 2. In this way we chose the most recognizable footstep sound that was judged as highly reproducible by the human body.

### Main experiment—task description

**Figure 1:**
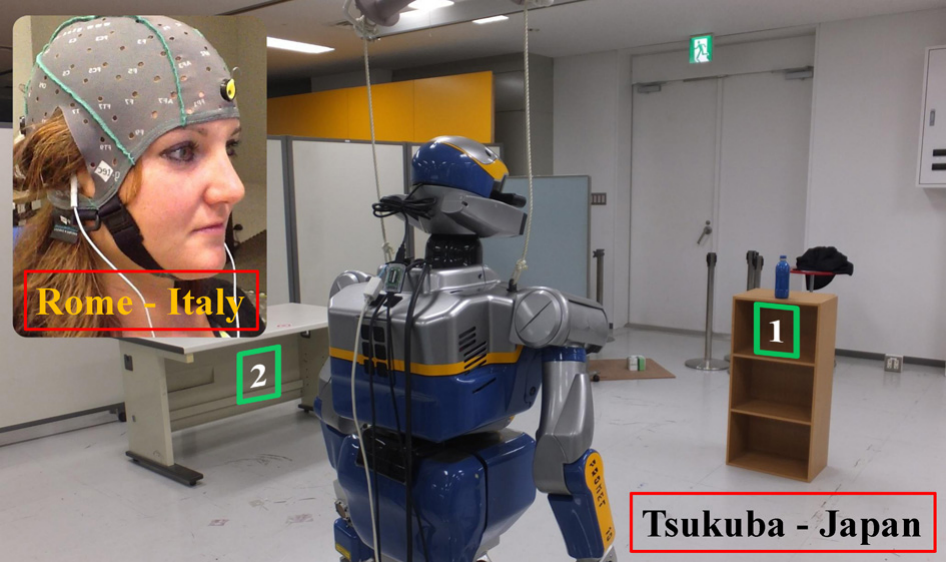
**Participants located in Rome (Italy) controlled an HRP-2 humanoid robot located in Tsukuba (Japan) by a SSVEPs-BCI system**. The subjects guided the robot from a starting position to a fist table (marked in green as “1”) to grasp the bottle and then drop the bottle as close as possible to a target location marked with two concentric circles on a second table (marked in green as “2”).

**Figure 2:**
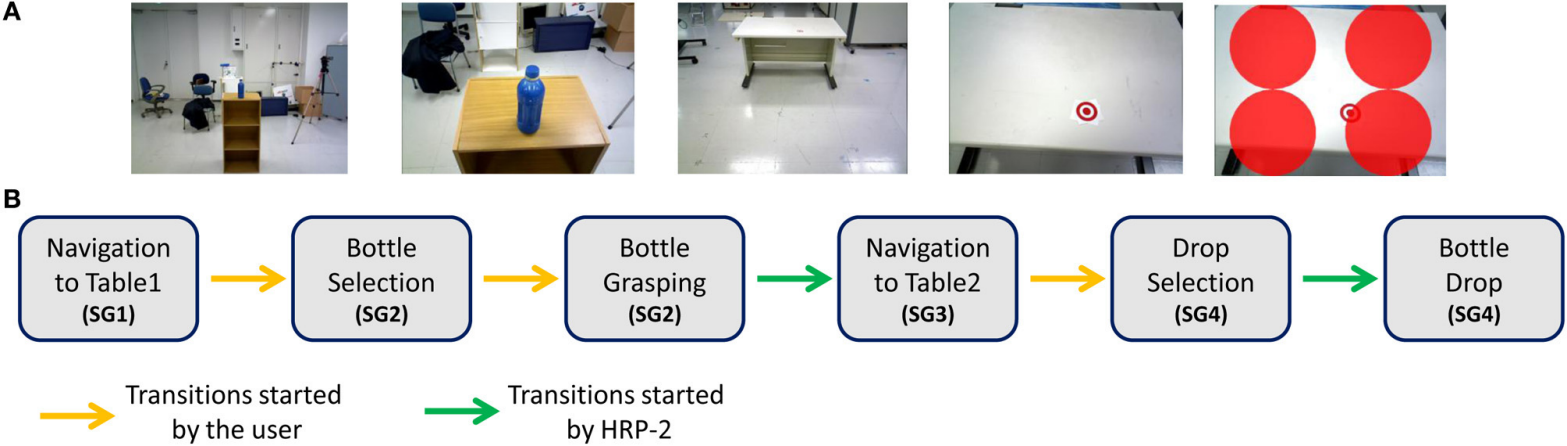
**(A)** A sequence of images depicting the different sub-goals (SGs). **(B)** A state-flow diagram showing the Finite State Machine (FSM). Yellow arrows represent transitions initiated by the user while green arrows represent transitions initiated by the robot.

Participants located in Rome (Italy) controlled an HRP-2 humanoid robot located in Tsukuba (Japan, see Figure 1) by a SSVEPs-BCI system. The task had four sub-goals (SGs). First the BCI user had to steer the robot from a starting position to a table (SG1) and command HRP-2 to grasp a bottle (SG2). Then, the participant guided the robot as close as possible to a second table (SG3) and tried to drop the bottle as close as possible to a target location marked with two concentric circles (SG4, see Figure 2A).

Participants were asked to complete the task as fast and accurate as possible. An enforced- (for all SGs, see section Enforced Selection) and a recursive-selection (SG4, see section Recursive Selection) were adopted to allow the user to complete the task. In half experimental conditions, we placed a mirror behind the second table to provide additional information about the spatial proximity between the table and the robot. During the task the BCI user heard a footstep sound either synchronous or asynchronous with the real footsteps of the robot (during SG1 and SG3) and could see or not the robot's body reflected in a mirror placed behind the second table (SG3 and SG4). In this way we had a 2 × 2 design with Footstep (Synchronous, Asynchronous) and Mirror (Present, Absent) as within subjects factors for a total of four experimental conditions. After each condition the subject answered questions concerning the experience they had (see questionnaire section Quality of the Interaction). Importantly auditory feedback was delivered in Italy. Data regarding robot's feet contact were streamed from Japan along with the video stream. Hence when a foot touched the ground we could deliver an auditory feedback to the user located in Italy synchronously or asynchronously with the video feedback.

### Procedure

The user was comfortably sitting in an armchair about 75 cm away from a 19” LCD screen operating at a refresh rate of 60 Hz upon which the user interface was displayed. The SSVEP classifier was trained on individual EEG data and a short video was presented to explain the goals to accomplish during the experiment. A trial consisted of the execution of the entire demonstration: from grasping to dropping the bottle on the second table. Two concentric circles indicated the target position on the second table. At the end of the trial, participants answered 4 questions to assess the quality of their interaction with the robot. An initial practice trial was performed before the experimental conditions. Note that we have been able to set the classifier error rate at 0% for all subjects. After training the error rate was null after 7.52 ± 0.22 s (mean ± s.e.m.). All participants completed successfully the training and were able to use the SSVEP.

### Data analysis

The Total time to complete the task, Walking time (i.e., time to steer the robot from the first table to the second one; SG3) and Place Accuracy (i.e., displacement between the target location and dropped bottle position; SG4) were used as measures of behavioral performance. We discarded from the main analysis one participant who did not follow task instructions and presented a Walking time 202% higher relative to the other participants. Thus, the final sample was of 8 subjects (2 female, 21.5 ± 1.06, range 20–23 years). An ANOVA with Mirror (present, absent) and Footstep sound (Synchronous, Asynchronous) as between subjects' factors was performed after checking for normality distribution (using the Shapiro-Wilk test).

Subjective answers to the quality of the interaction were analyzed by means of non-parametric tests Friedman ANOVA and Wilcoxon test for within-group comparisons.

### Hardware and software integration

#### Brain computer interface

EEG signals were acquired at 256 Hz by means of a g.USBamp (24 Bit biosignal amplification unit, g.tec Medical Engineering GmbH, Austria). We applied a band-pass (0.5–30 Hz) and a notch filter at 50 Hz. Eight Ag/AgCl active electrodes were placed on the POz, PO3, PO4, PO7, PO8, O1, O2, and Oz positions of the international 10–20 system. Fpz was used as ground electrode and the right earlobe as a reference.

#### Feature extraction

SSVEPs were extracted from the EEG signal using a minimum energy classifier (Friman et al., 2007). The system categorized 6 different classes with 100% accuracy in all participants after a short training (6 min). Five classes were implemented to steer the robot (forward, 6 Hz; backward, 9 Hz; turn right, 8 Hz; turn left, 14 Hz; stop, 10 Hz) and an additional zero-class detected when the user was not attending to any flashing stimuli. The frequencies were selected to avoid first or second harmonics and to minimize the risk of eliciting seizures in healthy subjects (Fisher et al., 2005). Critical frequencies fall in the range of 15–25 Hz. It is worth noting that some individuals may result sensitive also to higher rapidity of the flashing stimuli (Fisher et al., 2005). Moreover, frequencies higher than 25 Hz are difficult to render properly on a 60 Hz monitor. Thus, the selected frequencies of the flashing stimuli were 6, 8, 9, 10, and 14 Hz.

#### Graphical user interface

**Figure 3:**
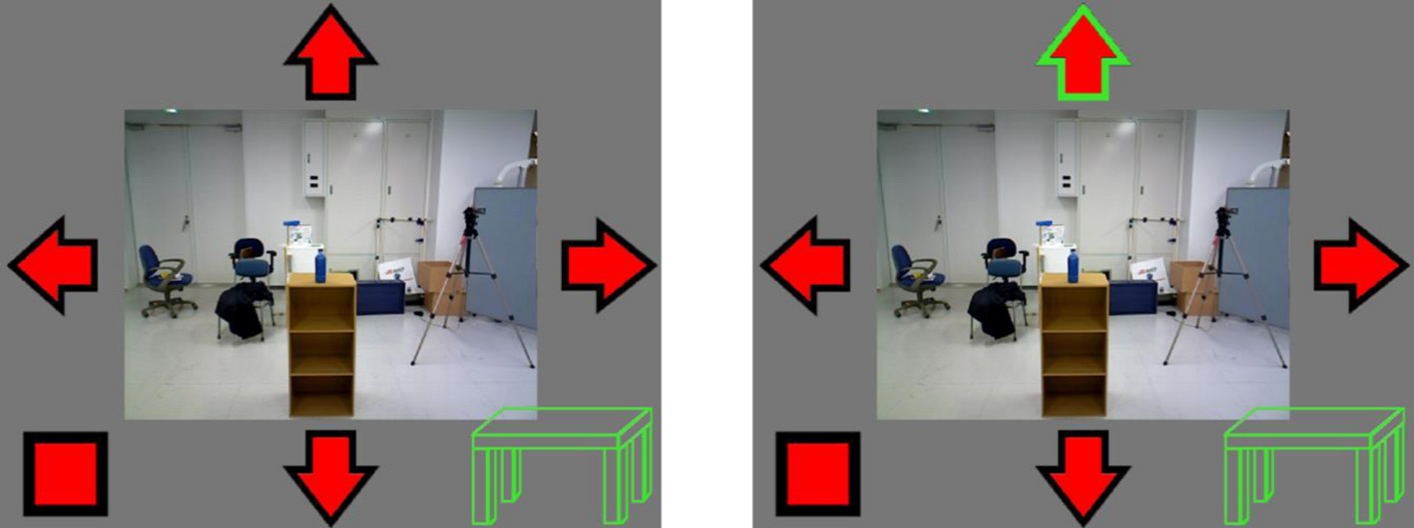
**Within the implemented graphical user interface whenever the BCI recognized a command (e.g., top arrow, forward-command), the corresponding icon's border changed color (from black to green)**.

During walking phases, four flickering arrows allowed the participant to send the robot instructions to move leftward, rightward, backward, or forward. A fifth flashing square was added in the bottom-left of the interface and was meant to stop the robot. When the BCI recognized one of these possible commands, the corresponding arrow or square's border changed color (from black to green, Figure 3).

From that moment, participants observed a change in robot's video feedback after nearly ~800 ms (~400 ms to send information to Japan, ~400 ms to receive robot's cameras feedback in Italy). Moreover, color-change signaled to the BCI users whether the system was correctly or incorrectly categorizing their intentions. This may be relevant for the experienced sense of control over robot's actions in addition to the audio-visual temporal matching. It is also worth noting that distal intention increases the sense of agency as measured by intentional binding (Haggard et al., 2002; Vinding et al., 2013). Finally a simplified table-icon changed color (from green to red) when the proximity of the robot to the table was too close and potentially dangerous (see Section HRP-2 Walking).

#### Enforced selection

Classification errors may interfere with robot's control if noise is introduced in the EEG data (e.g., the user gets distracted, makes involuntarily moves or has lapses of attention). Since SSVEPs' classification algorithm delivers a new output every 200 ms we adopted an enforced selection procedure to avoid conveying signals to the robot when the user did not want to (“Midas Touch” problem; Moore, 2003). This solution was meant for SG2 (grasping phase) and SG4 (dropping phase). Thus, with the enforced selection, a command was sent to the robot if the user held his selection for 2 s. This means that only after 10 equal and consecutive outputs the robot grasped or dropped the bottle. Based on our previous experience (in Gergondet et al., 2013 BCI-users had to held the command for 3 s) we reasoned that this solution could represent a good compromise between accuracy and performance.

#### Recursive selection

**Figure 4:**
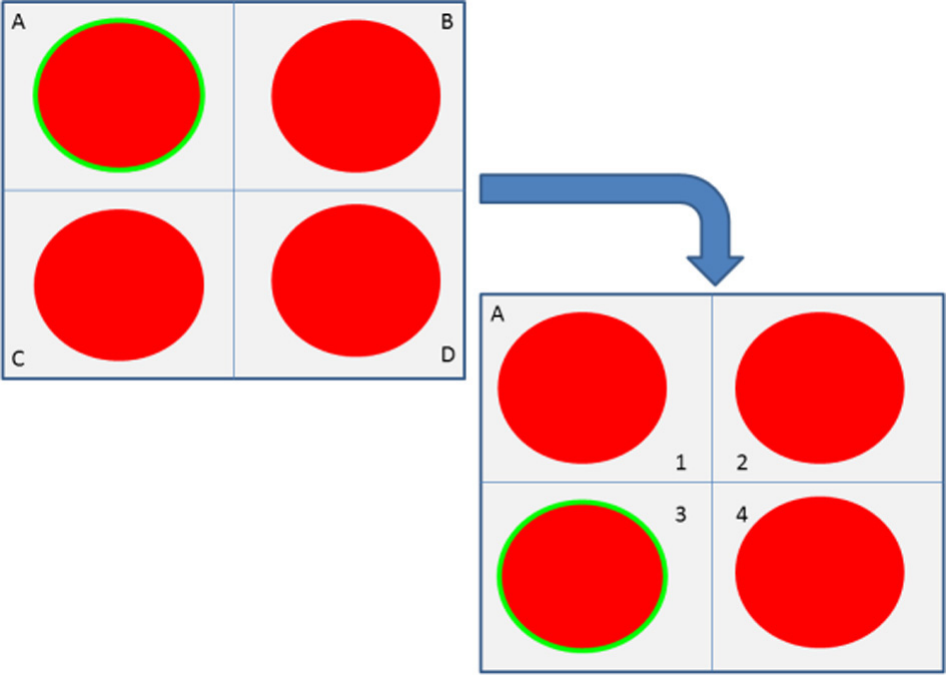
**Principle of the recursive and enforced selection during SG4**. For example the user first selects the “A” quarter which is then zoomed in to allow the subject to select the “3” quarter. The resulting final selection is “A.3.”

In the SG4 the user had to drop the bottle as close as possible to a target location. The dropping position was selected in two steps. The user selected one of four quarters then, the selected part was split again in 4 sub-parts. In this way the user had 16 alternatives to place the bottle. Importantly to reduce to number of errors (“Midas touch” problem, Moore, 2003) we coupled this recursive selection with an enforced selection (see section Enforced Selection; i.e., the user had to focus for 2 s on a stimulus before triggering a level change). The process is shown in Figure 4 and the four circles flashed at 6-8-14-10 Hz (upper-left and -right, bottom-right and -left, respectively).

#### Sub-goals transition

Transitions between different phases of the SGs were achieved by the implementation of a finite state machine (FSM, Figure 2B). In this way we combined both user' and robot's autonomy. The BCI user freely decided for the robot's walking directions, the moment to grasp the bottle and the place for dropping it. HRP-2 followed BCI-users' intention and autonomously implemented the correct movements for walking (SGs1-3, see section HRP-2 Walking), grasping (SG2, see section Robot's Grasping and Dropping Actions) and dropping (SG4, see section Robot's Grasping and Dropping Actions). Therefore, transitions between different SGs were triggered either by the user or the robot with a FSM as shown in Figure 2.

#### HRP-2 walking

The robot's head was slightly bent toward the ground (at a 25° angle) to allow the user to see upcoming obstacles. Participants performed a continuous control over robot's walking and the steering was possible trough the flickering arrows (see Graphical User Interface section). An Asus Xtion Pro Live was mounted on top of HRP-2's head. We adopted a Canny edge detection algorithm and a Hough transformation on the image to locate the corner of the tables in the depth map provided by the Xtion sensor. Given the intrinsic and extrinsic parameters of the camera we were then able to locate the position of the table within the robot's frame. As the robot approached the table it could be clearly seen in the depth map of the robot's camera and thus easily detected. A simplified table icon changed color (green to red) according to the proximity of the table (far or near). Overall we improved the safety and stability of the robot's walking by providing distance information to the BCI user and restraining robot's movements when it was too close to the table (i.e., avoiding accidental collision).

#### Robot's grasping and dropping actions

During SG2 (grasping) steering commands were not visible and the bottle, present within the field-of-view (FOV) of the robot, started flickering at 6 Hz. The user focused her/his attention on the bottle and instructed the robot to autonomously grasp it. The robot automatically repositioned itself according to bottle position and selected the arm to use. The arm followed a checkpoint trajectory close to robot's body. The robotic arm stretched and HRP-2 grasped the bottle avoiding possible collisions with the table. Then, after a small lift, the robot brought the arm back to its body up to the initial position through a checkpoint trajectory. During SG4 (dropping) we used an estimation of the height of the drop spot through the image provided by the RGBD camera mounted on the robot and implemented a soft-drop strategy by controlling the arm's speed using the algorithm described in Table 1. Both solution for grasping and dropping took advantage of HRP-2 capabilities to perform complex tasks and motor actions and highlight the coupling of software and hardware solution to combine both robot's and user's autonomy.

**Table 1:** **z estim, is the estimated height from the vision**.

**Algorithm for soft dropping**
**if** *in force > force threshold or z current < z min*
**then** Drop the object if a contact is detected or the hand has reached a low position
Open the gripper
*z speed = z speed*
**else**. Lower the speed command as the hand approaches the estimated contact height
**if** *z current > z estimate + 0.1* **then**
*z speed = z speed ref*
**else if** *z current > z estimate + 0.02* **then**
*z speed = z speed ref / 2*
**else**
*z speed = z speed ref / 10*
**end if**
**end if**

z current, is the current height of the robot's hand. z min, is the minimum allowed height, corresponding to the robot's physical limitations. z speed, is the speed command for the robot's hand. z speed ref, is a reference speed given before-hand. in force, is the force read from the wrist's force sensor. force threshold, is a force threshold defined before-hand obstacle detection during the phase where the user steers the robot to ease the control.

## Results

### Performance assessment

#### Total time

Data were normally distributed (Shapiro-Wilk test for all conditions: *p* > 0.20). The ANOVA did not reveal any main effect [all *F*_(1, 7)_ < 0.62, *p* > 0.45, η^2^ < 0.08] or interaction [*F*_(1, 7)_ = 4.53, *p* = 0.07, η^2^ = 0.39]. We performed an additional ANOVA to check any learning effect with the Order of the conditions as 4 level factor (Trial1, Trial2, Trial3, Trial4). We did not observed any learning effect [*F*_(3, 21)_ = 1.38, *p* = 0.27, η^2^ = 0.16].

#### Walking time

**Figure 5:**
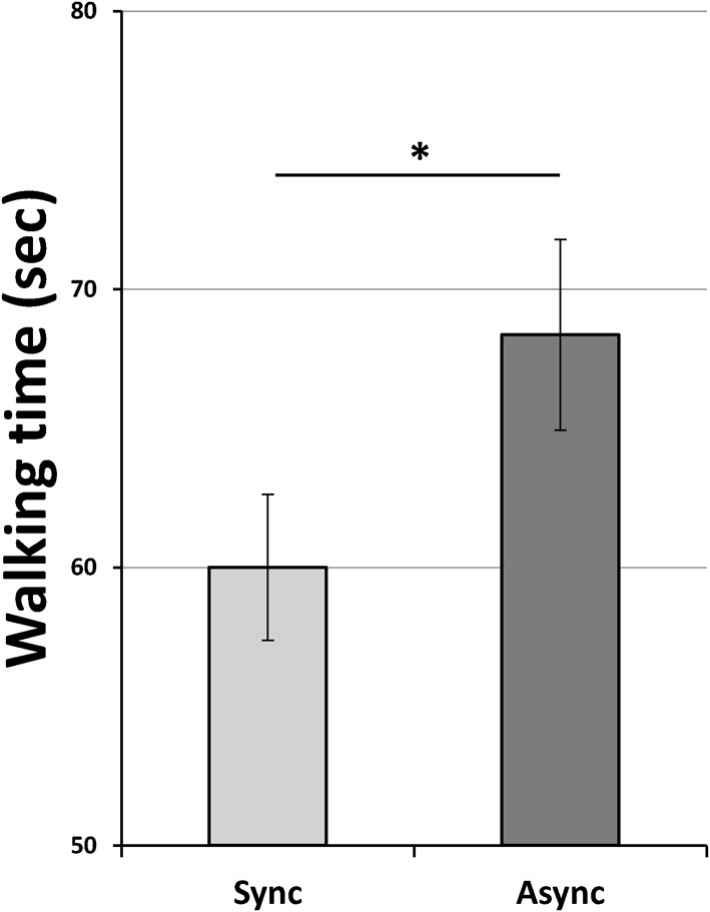
**Mean walking time to drive the robot from the first to the second table and drop the bottle**. Light-gray and dark-gray columns represent Synchronous and Asynchronous footstep sound heard by participants. Error bars represent s.e.m. Asterisk indicate significant comparisons (*p* < 0.05).

Data were normally distributed (Shapiro-Wilk test for all conditions: *p* > 0.15). The ANOVA revealed a main effect of Footstep [*F*_(1, 7)_ = 10.10, *p* = 0.01, η^2^ = 0.59] with faster time in the Synchronous (mean ± S.E., 60.00 s ± 2.62) relative to the Asynchronous condition (68.36 s ± 3.42; Figure 5). No main effect of Mirror [*F*_(1, 7)_ = 3.96, *p* = 0.09, η^2^ = 0.36] or interaction [*F*_(1, 7)_ z = 0.01, *p* = 0.97, η^2^ < 0.01] was found. We additionally checked for any learning effect. The ANOVA performed with Order as a 4 level factor (Trial1, Trial2, Trial3, Trial4) did not reveal any effect [*F*_(3, 21)_ = 0.86, *p* = 0.47, η^2^ = 0.11]. To rule out any role of distraction or drop of attention during the asynchronous condition we checked the total commands sent to the robot and the times the robot stopped (i.e., the BCI classifier categorized a “zero class”) as index of participants' uncertainty that may have explained the result.

Due to technical failure for one subject the number of commands and stops sent to the robot were missing in the Sound Asynchronous—Mirror Absent condition. The missing cell was substituted by the mean of commands and stops the subjects (*n* = 7) sent to the robot in that condition. Neither the number of sent commands in the Synchronous (25.87 ± 4.00) and Asynchronous (29.55 ± 3.00) condition [*t*_(7)_ = −1.53, *p* = 0.16, *r* = 0.06] nor the times the robot stopped in the Synchronous (4.44 ± 0.89) and Asynchronous (4.29 ± 0.67) condition [*t*_(7)_ = 0.27, *p* = 0.79, *r* = 0.28] did differ.

#### Place accuracy

Data were not normally distributed (Shapiro-Wilk test in two out of four conditions: *p* < 0.05). Non-parametric Friedman ANOVA did not reveal any differences in the Place accuracy (χ_3_ = 5.07, *p* = 0.16).

### Quality of the interaction

We assessed the perceived quality of the interaction by means of a questionnaire (Table 2). We assessed the felt agency (Q1) over robot's actions and no differences between experimental conditions (Friedman ANOVA, χ_3_ = 3.08, *p* = 0.38) were found. Throughout all the experiment, participants did pay attention to the images while they were controlling the robot (Q2, χ_3_ = 1.19, *p* = 0.75) and found easy to steer HRP-2 (Q3, χ_3_ = 3.65, *p* = 0.30). Importantly, participants reported no discomfort due to the flashing stimuli (Q4, χ_3_ = 1.26, *p* = 0.73) in any condition.

**Table 2:** **Participants answers to questions assessing the quality of the experience**.

	**Sound Sync**		**Sound Async**	
	**Mirror**	**no-Mirror**	**Mirror**	**no-Mirror**
Q1 I was in control of robot's actions	66.88 ± 6.74	73.13 ± 5.08	67.50 ± 7.07	71.88 ± 5.50
Q2 I paid attention to the images displayed	71.88 ± 6.40	71.88 ± 6.54	74.38 ± 8.10	77.50 ± 7.91
Q3 It was easy to instruct the robot about the direction where to move	64.38 ± 7.99	68.75 ± 5.49	65.63 ± 10.41	62.50 ± 5.00
Q4 Looking at the flashing arrows was difficult	25.99 ± 8.02	27.50 ± 7.96	28.13 ± 9.54	28.13 ± 7.44

Numbers represent values comprised between 0 and 100 (mean ± s.e.m.)

## Discussion

BCI systems based on MI, attentional selection (P300) or synchronization of activity in the visual cortex (SSVEPs) can be successfully used for walking and navigational purposes (for MI-BCI see; Leeb and Pfurtscheller, 2004; Pfurtscheller et al., 2006; Leeb et al., 2007a,b; for P300-BCI see Escolano et al., 2010; Curtin et al., 2012; Escolano et al., 2012; Choi and Jo, 2013; for SSVEPs-BCI see Bell et al., 2008; Prueckl and Guger, 2009; Diez et al., 2011; Choi and Jo, 2013; Gergondet et al., 2013).

Since the SSVEPs BCI system is a rather stable one, we adopted it to test the role of visual and auditory feedback during the remote control of a humanoid robot (HRP-2). More specifically, we explored whether temporal congruency of seen and heard input can modify the ability to steer a robot and the perceived quality of the human-robot interaction. We designed an easy way to use graphical interface sending to participants information about obstacles' proximity. Participants continuously controlled the walking directions of the robot by means of the SSVEPs flashing commands. The manipulation of audio-visual feedback was effective in modifying the participants' performance. More specifically, footstep auditory feedback delivered synchronously with the seen robot's steps allowed the participant to use less time for correctly driving the robot from the first to the second table. This effect was limited to the walking phase and did not generalize to the total time required to complete the task. It is worth noting that the overall time is composed of transitions autonomously initiated by the robot and that only the walking phase required an accurate control to turn and drive the robot to the second table. These factors may have flattened the overall time required to accomplish the task resulting in a non-statistical difference of the total time between the experimental manipulations. Importantly participants did not differ in the total number of commands and stops sent to the robot. This indicates that participants were not more imprecise or erroneous in sending commands to the robot in the asynchronous relative to the synchronous condition. The ability to efficiently decide when an action has to be performed within a given environment may be affected by the reliability of sensory information. Feedback uncertainty may indeed affect this decision making process (Wolpert and Landy, 2012). The sound of synchronous footsteps may have improved the ability to decide “when” changing command. In other words, the use of audio-visual synchrony may have helped BCI-controllers' decisions to better coordinate the robot. This result is relevant for current research in BCI the aim of which is to improve the human control over artificial devices either physical (e.g., humanoids robots) or virtual (e.g., avatars). Furthermore we showed that participants could maximize the performance (faster walking) taking advantage of audio-visual feedback. The observed advantage did not parallel any change in the perceived effort as indicated by the absence of differences in the questionnaire concerning the synchronous vs. asynchronous experimental conditions.

Our data expand a previous study that showed that background noise did not affect the users' BCI performance (Friedrich et al., 2011). Indeed the overall performance (total time and place accuracy) in the asynchronous conditions did not differ from synchronous conditions. Importantly, the increased ability to move the robot did not affect the dropping accuracy. Moreover the mirror did influence neither the speed nor the drop accuracy suggesting that spatial information from the mirror did not facilitate dropping ability. It is worth noting, however, that participants may need additional training or more time to actually learn to use mirrored images for a better control of robot's orientation respect to the external environment. Related to this, we also note that the relative narrow field-of-view (FOV) may indeed represent a limiting aspect of teleoperation (Gergondet et al., 2013). FOV can affect the ability to discriminate distances and consequently the ability of subjects to avoid obstacles and stop the robot at the right distance to perform an action. Moreover we applied recursive and enforced selection, a camera depth perception and combined user and robot's autonomy to facilitate the interaction with the environment. All these advancements were implemented in a graphical interface that allowed participants to pay attention to the environment while controlling the robot.

Overall we obtained two main results: (i) maintaining a good level of users' experience and (ii) improving the navigational performance. More specifically, the performance and quality of the interaction were assessed in this study involving eight healthy people who successfully teleoperated the robot through BCI. Importantly it has been reported the automatic tendency of the central nervous system to integrate tactile and auditory feedback even when presented with a slight temporal asynchrony (Bresciani et al., 2005). This may be in keeping with the result that in our setup asynchronous auditory feedback did not affect the perceived sense of agency. It is also worth noting that this result might also have been positively influenced by the visual feedback provided by coloring the selected command (see Graphical User Interface section). The combination of these two factors may explain why our participants maintained a good sense of agency throughout the task and experimental conditions.

Finally we did not observe a learning effect as previously reported (Gergondet et al., 2013) although the setups were different. Participants were not faster in the last trials relative the initial ones. This may indicate that the asynchronous auditory feedback may have disrupted a possible learning curve. Our results are relevant for current BCI research in that they highlight how congruent sensory information can improve human-machine interaction without affecting the quality of the experience.

### Conclusion

The continuous technological advancement in the field of controlling external devices introduced several possibilities to actually act on the environment through a surrogate. The advancement of peripheral devices made possible the combination of BCI and eye tracking technologies (Zander et al., 2010; Onose et al., 2012). Here we designed and tested an application that used SSVEPs BCI system to decode user's intentions. Future works should combine EEG and eye tracker systems to integrate robot's navigation and action to interact in the world. An example may be the use of eye gaze for navigation and object-integrated SSVEPs for action. This would increase the options to perform different actions on the same object. Eye tracker might indeed perform a rough recognition of user's navigational purposes and SSVEPs, through recursive and enforced selections, might categorize the action to be executed on a specific object. This line of improvement should also parallel the development of the same application scenarios with more immersive systems like head mounted displays and very immersive virtual reality experience of the type one can experience in CAVE systems. Moreover, more detailed questionnaire may shed new light about the feeling of control and comfort that remote BCI users may experience during similar human-robot interface. All in all, our data are important for designing user-friendly interfaces that allow people who cannot control their body anymore (e.g., spinal cord injured patients) to re-enter to the world in the most efficient possible way.

### Conflict of interest statement

The authors declare that the research was conducted in the absence of any commercial or financial relationships that could be construed as a potential conflict of interest.
